# A Treatise on Diseases of the Joints; with an Atlas of 16 Plates

**Published:** 1846-01

**Authors:** 


ART. VIII.

1. *Traité des Maladies des Articulations; accompagné d'un Atlas de* 16 *Planches.* Par A. BONNET, Professeur de Clinique chirurgicale à l'Ecole de Médecine de Lyon, &c. *Paris,* 1845.

*A Treatise on Diseases of the Joints; with an Atlas of* 16 *Plates.* By A. BONNET, Professor of Clinical Surgery in the School of Medicine of Lyons, &c. *Paris,* 1845. 2 vols. 8vo, pp. 1229.

2. *Recherches pour servir à l'Histoire des Tumeurs Blanches.* Par M. RICHET, Prosecteur de la Faculté, &c. (*Annales de la Chirurg. Français et Etrangère,* Mai, Juin, 1844.)

*Researches intended to elucidate the History of White Swellings.* By M. RICHET, Prosector to the Faculty of Medicine of Paris. *Paris,* 1844. pp. 146.

3. *De la Résection de la Tête du Fémur.* Par M. BONINO. (*Ann. de Chirurg. Franç. et Etrang.* Avril, Mai, 1844.)

*On Resection of the Head of the Femur.* By M. BONINO. pp. 38.

4. *De la Coxalgie.* Par M. le Docteur J. G. MAISONNEUVE, Chirurgien du Bureau central des Hôspitaux civils de Paris. (*Ann. de Chirurg. Franç. et Etrang.* Dec. 1844. Janv. Fev. 1845.)

*On Coxalgia.* By J. G. MAISONNEUVE, M.D., Surgeon to the Central Bureau of the Civil Hospitals of Paris.

5. *Recherches sur le Mal Vertébral de Pott. Mémoire couronné par le Conseil des Hôpitaux.* Par le Docteur TAVIGNOT. (*L'Expérience,* Juin, Juillet, Août, 1844.)

*Researches on Pott's Disease of the Spine.* By Dr. TAVIGNOT; *being a Memoir to which a Prize was awarded by the Council of the Hospitals of Paris.*

6. *Maladie des Articulations Costo-chondrales et Costo-vertébrales, avec ou sans Ramollissement Tuberculeux et Nécrose des Os du Rachis.* Par A. TOULMOUCHE, D.M., Professor de Pathologie externe à l'Ecole préparatoire de Médecine à Rennes. (*Gazette Médicale de Paris,* Janvier, 1845.)

*On Disease of the Articulations of the Ribs, with or without Tubercular Softening and Necrosis of the Vertebræ.* By Dr. A. TOULMOUCHE, Professor of External Pathology in the Preparatory School of Medicine at Rennes.

M. BONNET is already well known by numerous contributions to surgical literature in various journals, and by his work on Section of the Tendons and Muscles of the orbit; he now appears in a more ambitious character as the author of two large volumes on a most important class of diseases, the fruits—he informs us—of six years' diligent and special investigation, aided by the ample opportunities for observation which he enjoyed as surgeon-in-chief to the Hôtel-Dieu of Lyons. Both the character and position of the author, and the importance of some of the positions he advances, call for a somewhat extended notice of his work: but we shall not of course, attempt a systematic analysis of its entire contents. We shall limit our attention almost entirely to those points on which Bonnet's views claim to be, or really are, more or less novel and peculiar; and we shall take occasion to advert to whatever seems most worthy of notice in the several memoirs whose titles are above transcribed.

Few authors now-a-days think it necessary to offer any excuse for adding to the already unwieldy bulk of medical literature, but M. Bonnet explains at great length his motives for writing the present work, and his explanation may be reduced simply to this:—That no complete treatise on diseases of the joints has hitherto appeared. It is true that Brodie has published what M. Bonnet rather superciliously describes as "a collection of memoirs on some very limited points of pathological anatomy, and of diagnosis, under the name of a treatise on Diseases of the Joints;" but we have no work "qui embrasse l'ensemble des arthropathies;" and it is this deficiency which our author professes to supply. All preceding writers have, he maintains, taken a one-sided view of the subject. One set of authors, belonging to the school of vitalism, have chiefly investigated the relations of diseases of the joints with general affections of the system, and another, the pathological anatomists, have almost exclusively examined their local causes, lesions, and treatment. M. Bonnet's aim has been to combine the somewhat exclusive tendencies of those two schools, "to apply the idea so frequently spoken of, but so seldom followed out, of not separating medicine and surgery." (p. 197.)

With the view of considering diseases of the joints under every aspect, M. Bonnet divides the work into three parts. The first part is devoted to diseases of the joints in general, and is divided into four chapters:—*Pathological Anatomy*, *Etiology*, *Diagnosis*, and *Treatment*. The second treats of the several kinds of diseases to which joints are liable. And in the third are considered the special characters and treatment of those several diseases as they occur in each particular joint. This arrangement, whatever may be its advantages, has the inconvenience of leading to frequent repetitions, and as frequently severing matter which would be much better continuously embodied.

We may here observe that M. Bonnet limits himself to the consideration of what he terms "vital disease,"—"organic alterations of the joints." "Traumatic lesions," however, "such as sprains, contusions, and wounds, are studied, but chiefly in their relations to the pain, inflammation, and abcesses which they may cause." (vol. xi. p. 1.) The history of fractures and dislocations of the joints, and of those deformities which are not consecutive to diseases of the articulations, such as club-foot, congenital luxations and lateral curvature of the spine, is omitted to avoid extending the work to three volumes.

I. Pathological anatomy of the joints. M. Bonnet states that on this subject his *dissections* have not led him to the discovery of anything new: "If," he says, "I have added anything to the pathological anatomy of the joints, it is less by the ordinary means of dissection than by employing methods of research imperfectly or seldom applied . . . . . with this view I have had recourse to the chemical analysis of the diseased products, to the artificial production on the dead body of various physical lesions, and to the comparative study of diseases of the joints in men and in animals." (p. xi.) We shall therefore only notice those points on which M. Bonnet claims to have thrown new light, except when reference to other matters is necessary to introduce or explain our author's views.

*Chemical analysis.* The views to which M. Bonnet has been led by the chemical analysis of the products of disease may be thus stated: The elementary lesions which occur in the structures of the joints, whether in the bones or in the soft parts, are divisible into two classes: one comprising such products, *e. g.* pus and tubercle, as are completely insusceptible of organization, and which, being therefore only capable of cure by elimination, tend, in the vast majority of instances, to make their way to the surface, and determine the successive absorption of the textures in which they are deposited; the presence of these products is consequently unfavorable, and generally indicates an unmanageable state of disease. To this class we need not further allude. The second class includes those products that are capable of being organized: they are "*fungosities,*" and *lardaceous, fibrous, cartilaginous,* and *bony tissue,* which all essentially consist of *plastic lymph* in one or other of the phases of its development. Under ordinary circumstances, plastic lymph, at first unorganized, is soon penetrated with vessels, and ultimately becomes cellular or fibrous, or even cartilaginous or bony; but it by no means uniformly runs through its regular periods of organization, which on the contrary may, under the influence of various disturbing causes, be more or less permanently arrested at an early stage of its development. Thus, plastic lymph, when once organized, is subject to the diseases of the natural textures; for example, it may become infiltrated with pus or with tubercle; its organization then sustains a true arrest of development, and it remains in a state of which the granulations of an ulcer, and "*fungosities*" of joints are examples; a similar effect may be produced by a purely local cause, as the presence of a foreign body; in illustration of which M. Bonnet refers to the granulations in necrosis or of an issue, which persist indefinitely until the dead bone or issue peas are removed, when they pass to their third stage of cicatrization or conversion into fibrous tissue. In proof of these views, M. Bonnet appeals to the chemical analysis of *fungosities* and of *lardaceous tissue. Fungosities* he has found by chemical analysis to "be formed of serum and of fibrin, permeated by vessels; in a word, they consist of plastic lymph at that stage which I have considered as the second period of its organization." (vol. ii, p. 19.) *Lardaceous* tissue, which, together with *fungosities* and pus, forms most of the alterations known as white swelling, including the disease described by Brodie as a "morbid change of structure of the synovial membrane" is proved by chemical examination to consist of fibrous and cellular tissue, infiltrated with serum and with fibrin; if the former preponderate, it is soft; if the latter, it is white and compact; occasionally it is permeated by an unusual number of vessels, and is then less dense, and more or less red. *Lardaceous* tissue may be considered as intermediate between *fungosities* and *fibrous* tissue, and thence, both in theory and in fact, its presence indicates a better state of the constitution than the existence of *fungosities* alone does, and when the tumour contains both, the more the former preponderates the more favorable is the case. It is unnecessary to notice M. Bonnet's remarks respecting adventitious bone and cartilage; and as regards *fibrous* tissue, we need merely state that M. Bonnet regards it as the indispensable condition of recovery from the above-mentioned lesions, and that is found in every case which either tends to a cure, or has actually been cured, excepting where *fungosities* are developed in bones, and osseous deposit has occurred in the organized lymph.

It might seem natural here to inquire, what are the circumstances which modify the products of disease in affections of the joints? why in some instances products incapable of organization are generated? and still more, why it is that when an organizable product is effused, its organization is in some cases arrested, and in others completed? But M. Bonnet's views on those points will be better considered when we come to his chapter on etiology.

*Morbid changes.* Such are the conclusions which M. Bonnet has drawn from the chemical analysis of the products of disease. He does not attempt to trace or describe the anatomical characters of the morbid alterations in the various stages of their progress, and indeed he is of opinion that this mode of research has been nearly exhausted by previous inquirers. M. Richet, however, in the memoir mentioned at the head of this article, endeavours to trace minutely throughout their successive periods the pathological alterations caused in the joints by inflammation, and thence to establish the following conclusions: 1st. That neither cartilage of incrustation, or extra or intra-articular fibrous tissue are susceptible of primary alteration. 2d. That consequently disease of a joint can only commence in the synovial membrane, or in the bone, excepting in the rare cases in which it begins in the soft parts external to the joint. And, 3dly. That the diseases termed white swelling are always the result of *synovitis* or osteitis, except in the comparatively very few instances in which they are consequent on tubercular degeneration. (pp. 174-5.) The last of those conclusions, though differently expressed, substantially agrees with M. Bonnet's views, and in other respects also we shall find the two writers pretty nearly agreed, although their inquiries were so very differently conducted.

*Synovitis.* Though dissections of joints affected with *chronic synovitis,* in its advanced period, are numerous, opportunities of examining the anatomical alterations of *acute synovitis* in all its stages are rare, and M. Richet therefore bases his description on numerous experiments performed on animals, and on some cases of traumatic arthritis, dissected at various stages of the progress of the inflammation. Within from four to six hours after having freely exposed the synovial membrane of a dog, sanguineous congestion commences in the sub-synovial cellular tissue, rather than in the membrane itself. In about ten hours the membrane loses its polish, but M. Richet never found it become preternaturally *dry.* During the second day the redness becomes more superficial, and less uniform, being disseminated in patches, and the surface of the membrane is covered with a sero-sanguineous oozing, which gradually gets thicker, and by the third is replaced by thin pus: the surrounding tissues are now highly congested, the synovial membrane is uniformly red, and very minute granulations appear on its surface. The following days all those appearances become more marked, and from the fifth to the twelfth day, a pseudo-membranous exudation forms on, and becomes intimately adherent to the granulations above mentioned: and at a still later period the synovial membrane assumes a fungous appearance, and forms a prominent chemosis round the cartilages, which retain their natural whiteness. In the case of a man who died of purulent arthritis on the twenty-fifth day after the removal of a foreign body from the knee, numerous soft fungous granulations formed a prominent ridge round, and encroached on, but did not adhere to the cartilages, which were intact. On the sixty-third day, after pure alcohol had been injected into the joint of a dog, the chemosed synovial membrane, covered with the pseudo-membranous exudations almost completely covered, but did not adhere to the cartilage, which had sustained no alteration, except a slight loss of polish, and an obvious diminution of its thickness. Such were the appearances observed up to about the sixtieth day. The changes that occur subsequently, M. Richet describes from pathological specimens, taken from the human subject; for though a white swelling, when amputated, has always existed more than two months, yet when the disease has commenced in the bone, as the synovial membrane is secondarily affected, opportunities occur of examining it in almost every stage of inflammation, which in such cases is not unfrequently chronic from the commencement. When inflammation of the synovial membrane, then, is very protracted, the granulations on its surface become more developed, and greatly increase its thickness, which is also augmented by œdematous infiltration, externally in œdema, which generally soon passes into the induration termed *lardaceous*. The natural structure of the membrane is now often completely lost and confounded with the new deposition on both its surfaces, but sometimes the adventitious structure can be removed, and the synovial membrane demonstrated. As already stated, the synovial membrane at first forms a prominent ring round the cartilages, but gradually it, or rather a pseudo-membranous prolongation from it, encroaches on, and more or less completely covers their surface, to which at first it is not adherent, but soon becomes so, feebly at first, after a time more firmly. The cartilages, during this process, become thin, and if they are eroded, the synovial membrane implants parasitic roots on the denuded bone. The cartilage being then included between the bone, (become highly vascular,) and the synovial false membrane, is slowly absorbed. Generally, when the disorganization has gone a certain length, pus or other fluid is effused into the joint. But in some the synovial membrane is greatly thickened and altered with very little fluid in the joint: this is the state termed *fungus articulorum*, the disease described by Brodie as a peculiar morbid state of the synovial membrane, which M. Richet maintains, from having traced up the successive alterations in various cases, is simply an advanced stage of very protracted chronic synovitis. When *fungosities* are once formed, they do not seem capable of retrogression; if the disease is arrested, they become firmer, and their cure consists in becoming truly indurated. (pp. 31-43.)

*Osteitis.* The anatomical characters of osteitis, especially in its early stages, are not very accurately determined, because an opportunity of examining it is not of frequent occurrence; we shall, therefore, give an abstract of M. Richet's observations, which relate entirely to chronic osteitis of the articular extremities of the long bones, an affection apparently often connected with the scrofulous diathesis, and thence, perhaps, often regarded as a scrofulous affection of the bone.

We must first refer to a point in the anatomy of the long bones, to which M. Richet attaches much importance, viz. the free communication that exists between the cancellous texture of both extremities of a long bone through the medium of the medullary canal. In proof of this communication, M. Richet adduces two experiments,—1st, If a recent long bone is suspended vertically, after the removal of a portion of the compact layer of its depending extremity, the oily matter will exude entirely even from the cancellous texture of the upper extremity; 2dly, Water injected into the upper extremity of a femoral bone similarly prepared, escapes below in a few seconds. This fact, M. Richet considers, not only explains the easy propagation of disease from one part of a bone to either extremity, and the pain often experienced along and at the extremity of the bone opposite to that which is diseased, (p. 11,) but also gives a valuable diagnostic sign, inasmuch as when such pain does occur, he concludes that the bone must be the seat of the disease. (p. 153.) Now, though inflammation is the disease to which bones are most liable, and their spongy extremities are most frequently affected with it because of their great vascularity, yet the progress of inflammation in bone is so slow, that we can seldom examine it at its commencement, at least in the part originally affected; but in consequence of the facility with which it is occasionally propagated from one to the other extremity of a long bone, M. Richet has several times been able to examine the early stage of inflammation at one end, secondary to a more advanced stage at the other. In such cases, the bones, clothed with a thickened periosteum, seemed red and vascular, and, on tearing off that membrane, minute drops of blood, reappearing several times when wiped off, exuded from the surface of the bone, which sometimes, but not constantly, is decidedly enlarged, even at this early period. On sawing the bone longitudinally, it is found to be evidently softened, the cut surface is of a wine-red colour, and the cells, which are enlarged and filled with blood, mixed with oily matter, sometimes yield under the finger, sometimes are preternaturally condensed. At a more advanced stage, the foregoing appearances are exaggerated, and the compact tissue presents numerous minute perforations for the passage of vessels into the bone, on whose surface periosteal secretions may now begin to be deposited, whence, as well as from the expansion of the cells, its bulk is augmented. M. Richet especially insists on the enlargement of the bone, which has been denied by several English authors, but which he has repeatedly verified by dissection. He conceives also that it can be infallibly detected in the superficial joints during life by pressing on opposite points of the condyles of the femur, for example, firmly with the finger and thumb, until the œdema of the soft part is removed; the diameter of the bone can then be taken, with callipers placed in the depressions left by the fingers, and compared with that of the opposite bone. M. Richet considers that the enlargement so detected is a certain proof of the bone being diseased, and may also aid in determining whether it is primarily or secondarily affected, as the enlargement is greater, and occurs more rapidly in the former than in the latter case. (p. 154.) Without in the least disputing the accuracy of M. Richet's dissections, we think the mode of mensuration he recommends eminently calculated to lead us to an erroneous conclusion, and we think his confidence in its accuracy has led him to regard enlargement of the bone as more frequent than it really is: that it does occur, and that not very rarely, has been long perfectly known; but we owe to M. Richet a more minute account of the mode of its development.

Proceeding with the further changes effected by inflammation in bone, we find that the redness, at first uniform, at length becomes concentrated at certain distinct points, varying from the size of a pea to that of a small nut, in some of which coagulated blood is found effused into the cells. At this period the compact layer of bone interposed between the cancellated texture and the cartilage of incrustation, becomes thin and perforated, giving passage to dark blood, which detaches the cartilage already preternaturally thin, and commencing to be pierced with holes beginning on its attached surface; but frequently the separation of the cartilage is effected by granulations (*fungosities*) growing from the diseased cells, and then, on removing the cartilage, the compact layer of bone comes away with it, and the granulations appear as a red fungous, uniformly flattened mass, because of the resistence offered to their development by the compact texture of the bone. That the granulations by which the cartilage is detached spring from the spongy cells, and not from a layer of cellular tissue, or of synovial membrane, interposed between the cartilage and the bone, as MM. Gerdy and Blandin, together with Mr. Liston, respectively maintain, is shown by the fact, that on tearing off the cartilage its attached surface is always rough from portions of bone adhering to it; the granulations, therefore, not being denuded until a film of bone is removed with the cartilage, must be situated beneath and not between the two structures. Concrete pus next forms in, or round, the isolated depôts of blood already mentioned, pronounced by M. Cruveilhier, in the specimens shown to him by M. Richet, to be true abscesses of bone; and the cells, in those situations, either become necrosed, or are absorbed, leaving a cavity of corresponding size; occasionally the sanguineous infiltration is not collected in circumscribed deposits, but is uniformly diffused; a large portion of the bone may then be necrosed, and either isolated by new bone, or the sequestrum may project into the joint. When the cartilage is perforated, the joint becomes diseased, and the inflamed synovial membrane undergoes the alterations already described. Finally, in some rare cases, the granulations of the bone detach the cartilage *en masse*, and it is found lying in the joint reduced to a very thin film. It is in the advanced stage of osteitis that the inflammation frequently extends to the other extremity of the bone, through the medullary canal, which is found intensely red throughout its entire length, though externally the shaft of the bone exhibits no indication of disease whatever. Osteitis also occurs as an affection secondary to synovitis, and the changes are essentially the same, but slower in their progress, and there is generally an early and considerable superficial deposit of bone near the joint. (pp. 130-151.) M.

Richet attempts no explanation of the fact that bone is sometimes softened, and sometimes rendered denser by inflammation; in other words, that the parietes of the spongy cells are sometimes thinned, and sometimes hypertrophied. M. Bonnet accounts for this on the general principle already explained. Plastic lymph in bone, as in other textures, tends to become fully organized, and here the last stage of organization is conversion into bone: but here, as elsewhere, its development may be arrested, and if it be completely so, it remains in the state of *fungosities*, which cause the softening and absorption of the tissues they are in contact with, and this happens in patients whose constitutions are greatly deteriorated; but when a certain degree of vigour and reaction exists, the arrest of development is only partial, more or less of the effused lymph becomes ossified, and the bone is proportionably condensed. The same considerations, M. Bonnet thinks, clear up the much-disputed question whether tubercles cause softening or induration of the bone, in which they are deposited: when the bone is hardened, it is not attributable to the deposition of tubercle, but to the contemporaneous secretion of plastic lymph, which has gone through its regular stages of organization. (vol. i, pp. 34-6.)

We may here just remark that M. Richet's description of osteitis includes the disease described by Brodie as "a scrofulous disease of the joints."

*Diseases of cartilage.* M. Richet next considers the pathological anatomy of the cartilages of incrustation. Both he and M. Bonnet adopt the opinion that cartilage is not vascular, and is not covered by the synonial membrane. We shall not detail M. Richet's arguments to show that cartilage is, to use his phraseology, or rather that of M. Velpeau, "organic, but unorganized," as they are only those previously urged by many whom he does mention, and by Toynbee, whom he does not mention. Suffice it to say, that M. Richet considers cartilage analogous in structure to the epidermis and the nails, and that it derives a parasitic nutrition by imbibition or endosmose from the bone to which it is intimately and *immediately* attached, and from the synovia with which its free surface is bathed. In evidence of the facility with which cartilage imbibes synovia, M. Richet refers to the fact, observed by Bichat, of it and the synovial fluid having been found yellow in jaundice; and to experiments, in which he injected various coloured fluids into joints of animals, and shortly after (within ten minutes in one experiment) found the cartilage uniformly stained throughout its entire thickness, while the other textures were only superficially coloured; and that the liquid part of the blood is imbibed by cartilage from the bone is shown, M. Richet considers, by the separation and erosion of the cartilage in disease of the articular extremities of the bones, which he attributes to this element of their nutrition being perverted or cut off. Consistently with these views, M. Richet denies that cartilage is susceptible of inflammation, or of active participation in the diseases of the adjacent tissues.

The only direct proof that cartilage is susceptible of inflammation, would be to demonstrate its vascularity; and it is singular, M. Richet says, that long as the matter has been in dispute, no preparation showing vessels in cartilage has yet been produced. Vessels belonging to the membranous prolongation of the inflamed synovial membrane, have been erroneously considered as evidence of cartilage becoming vascular on the surface; and the punctated redness caused by minute perforations of cartilage being filled with blood from the cancellated texture of diseased bone, has been mistaken for vessels penetrating the cartilage from the bone. But M. Richet, in addition to this negative argument, urges that cartilage never becomes vascular under circumstances in which it must be expected to do so, were the occurrence possible. By no description of physical or chemical injury could he excite a trace of vascularity in the cartilages of animals. In man also accidental solutions of continuity of cartilage remain unaltered, and without any attempt at repair, for he recently found in a patient who died a month after having fractured the lower extremity of the radius, the fracture perfectly united, and all the adjacent textures highly vascular, but the cartilage, which had been divided, as the fracture ran into the joint, showed no vascularity or other alteration; the edges of the division were like that of a nail cut with a scissors. When cartilage is exposed, as M. Richet once saw after disarticulation of the knee, and as has occurred in several other cases, it neither becomes red nor inflamed, but is detached—pushed off as it were—by granulations from the bone. Again, portions of cartilage are not unfrequently met with in joints in a very advanced state of disorganization, retaining all the chemical and physical characters, but somewhat rough and thin, showing that their absorption had commenced, but not manifesting the slightest trace of vascularity. And, finally, in cancerous disease of a joint all the structures become involved, excepting the cartilages only, a fact long since observed by J. L. Petit, and of which M. Richet gives a new example.

As M. Richet denies any active participation of cartilage in disease of the joints, it remains for him to account for those changes which they undeniably do sustain; and these he also refers to perversion of their nutrition, or to mechanical influences. And seeing that every alteration of cartilage, even its total disappearance, *may* occur in joints, not indeed healthy, but certainly manifesting no sign of serious disease, he urges that when the same alterations occur in white swelling, they must be a consequence not the cause of the disease.

In joints, otherwise apparently healthy, and which performed their function naturally, and were free from pain during life, the cartilage may present various alterations. The slightest change seems to be *loss of elasticity*, first noticed by Delpech, which is usually accompanied by, and is, perhaps, the first stage of *softening*, in which the cartilage has lost its polish, and presents the appearance of parallel fibres implanted perpendicularly on the bone, and adhering through the medium of a firm transparent, gelatinous substance, or without any lateral connexion, and in either case flexible under the finger. M. Richet has repeatedly seen this condition of cartilage in old subjects, and has always found it coincide with a diminished quantity of synovia in the joints; and, coupling those two circumstances, he refers the change to the nutrition of cartilage by the synovia, and from the bone becoming less active. We consider it as somewhat analogous to the senile alterations, and fall of the teeth and of the hair. Partial or total absorption of the cartilage, is perhaps, the third stage of this alteration; at all events it may occur without symptoms, and in joints in other respects healthy, at least apparently so; of which M. Richet saw a remarkable example in the ankle and knee-joints of an old man, a patient of M. Velpeau's, who walked freely a few days before his death, and during life never complained of any affection of the joints. Indeed, eburnation of the articular surface of a bone with absorption of the cartilage, is not unusual in old persons, and sometimes is attended with trifling inconvenience; and Girard, (Archiv gén. de Méd. 1824, t. iv, p. 195,) and Dupuy, (Id. 1835, t. ix, p. 276), have ascertained that the same condition exists in the neck of the astragalus of most old draught horses, without disenabling them to work.

Whenever cartilage is rough, uneven, or chiseled, there is always, M. Richet observes, acute or chronic disease of the joint, and those alterations are due to wearing with absorption, acting chiefly on certain points, and always caused by perverted or defective nutrition consequent on disease of the bone, or the presence of an abnormal liquid in the joint; for as cartilage lives at the expense of the bone and the synovial fluid, if they are diseased, it suffers also. In like manner, *erosion* of cartilage (for M. Richet rejects the term *ulceration* as applied to that structure) is always a secondary affection, sometimes depending on disease of the synovial membrane, but much more frequently, M. Richet thinks, on disease of the bone. In the latter case the erosion is almost always accompanied with more or less inflammation of the synovial membrane and effusion into the joint; and such are the cases, M. Richet says, which have been mistaken for primary ulceration of cartilage; but, he adds, the bone is *always enlarged*, and if sawn longitudinally *through the centre* of the erosion, a vascular inflamed point will be found corresponding to each erosion, while the bone in the interspace may retain its natural texture and colour. "Nevertheless," M. Richet exclaims—

"Many surgeons, Brodie and others, maintain that those losses of substance are the result of primary inflammation of the cartilage, of ulceration which subsequently extending to the adjacent structures, give rise to white swelling." (p. 168.)

This position, M. Richet says, Brodie founds on several imperfect, and therefore valueless, observations in which the only alterations found on dissection are stated to be "ulceration" of the cartilages, with enlargement ("*gonflement*") of the bone, and our author regrets that Brodie did not take a hint from this "*gonflement*," and examine the interior of the bone, which he would have infallibly found presenting signs of an advanced stage of inflammation. M. Richet also appeals to the absence of pus, in some cases as an evidence that erosion of cartilage is not ulceration, an objection which, he says, Brodie has endeavoured to anticipate by a petitio principii,—by assuming that ulceration of cartilage differs in this respect from ulceration of soft parts.—In fine, M. Richet concludes,

"1st. That cartilage of incrustation may be more or less altered in its structure.

"2dly. That its alterations are never inflammatory, and that consequently the terms *inflammation* and *ulceration* of cartilage should be rejected and replaced by the phrases *softening*, *wearing*, ("usure,") and *erosion*, &c.

"3dly. That the alterations are very seldom primary, and rarely occur, except the synovia or the bones have sustained modifications, which, by perverting the nutrition of the cartilage, leave them subject to the action of chemical or physical influences." (p. 174.)

In stating M. Bonnet's and M. Richet's views we have made scarcely a comment on them, and the length to which we have gone will prevent our making as many as we had intended ; a few are, however, necessary. In the first place, we must observe that M. Richet, though he criticises Brodie's cases and doctrines, is either very imperfectly acquainted with, or very careless in his exposition. Brodie expressly says that ulceration of cartilage may arise from several causes, of which he mentions as the chief, disease of the synovial membrane, chronic inflammation, and scrofulous alteration of the bone, and "a morbid condition of the cartilage itself;" yet M. Richet writes as though Brodie considered ulceration of cartilage to be always a primary affection of that structure. M. Richet also reproaches Brodie with not having examined the bones sufficiently, and indeed saying nothing about them, except that they were *enlarged;* now, it is remarkable that there is no mention of the bones being enlarged in *any one* of the thirteen cases recorded in Brodie's chapter "on the ulceration of the articular cartilages;" but if M. Richet had read those cases, he would have found evidence that Brodie did examine the bones, and he would also have found evidence confirmatory, so far as it goes, of his own views. Of the thirteen cases eight only are given as examples of primary, and in *all* of them, except the first, (case 26,) it is expressly said the denuded surface of the bone was diseased; (carious or ulcerated being the terms used;) it is no doubt added that the bones retained their natural texture and hardness, except indeed in the 38th case, in which a considerable extent of the cancellous structure is stated to have been diseased, whence it is to be inferred (but it is an inference only) that in the other cases the cancellous structure was unaltered. We do not indeed quite understand this account; caries is a condition in which the bone presents increased vascularity, extending some distance, beyond the carious part, together with softening and suppuration ; yet in several of the cases, e.g. the 28th, we are told that the caries was unaccompanied with the formation of pus; besides, caries particularly affects the cancellated structure of bone, but here it is stated to be confined to the thin layer of compact tissue on the articular extremity of the bone, without the vascularity, and the rarefaction, and softening of that tissue essential to its existence. It is difficult of course to draw any conclusion from those cases beyond the negative, that they certainly do not prove the primary ulceration of cartilages. On the contrary, they point directly the other way.

But if M. Richet has misunderstood one English writer, he has completely overlooked another ; and yet his description of the invasion of cartilage by the synovial membrane, or pseudo-membranous prolongations from it, might seem to be in a great measure borrowed from Mr. Key. We cannot indeed affirm that such is the case, for M. Richet has evidently seen what he describes, and may have had no knowledge of Mr. Key's labours ; whether this be the case or not, M. Richet's account is valuable, as tracing the progress of the alterations of the synovial membrane in its various stages. Key and M. Richet both agree that cartilage may be absorbed through the instrumentality of the synovial membrane, and the chief difference between them is, that Mr. Key thinks this is effected in chronic synovitis, by the gradual development of the synovial membrane itself, and in acute cases by a false membrane, effused from, and superadded to, the synovial membrane; whereas M. Richet has, in every case, whether acute or chronic, seen the encroachments on the cartilage effected by the formation of a false membrane. The views of Mr. Key and of M. Richet respecting cartilage and its alterations, are somewhat analogous, but yet different. Key considers that the organization of cartilage is very low, M. Richet denies that it is organized at all. Mr. Key says its primary alteration is a breaking up of its tissue, and M. Richet says pretty much the same thing; but Mr. Key considers that this primary breaking up is a vital action originating in the cartilage itself, and seems to imply, without directly saying so, that this vital action is inflammation, while M. Richet attributes the alteration to the nutrition of the cartilage being impaired or cut off by the imperfect supply or obstruction of the fluids that feed it. Mr. Key thinks that disorganization of cartilage, occurring independently of disease in the other structures of the joint, must lead to suppuration, from its disintegrated particles mixing with the synovia, and irritating and causing inflammation of the synovial membrane; while M. Richet maintains an opposite opinion. Mr. Key, indeed, subsequently modifies his statement, admitting it to be possible that the debris of cartilage may be reduced to an homogeneous fluid, mix with the synovia, and be absorbed. And that such is the case, M. Richet shows, as cartilage may be *softened*, reduced to a *fibrous state*, or even *totally disappear* in joints, which certainly cannot be called healthy, but which sometimes present little or no symptoms of derangement during life.

We have seen that M. Richet denies that cartilage ever becomes vascular, and how he explains the alleged examples of such an occurrence. We do not pretend to be able to determine this question. When such competent observers as Sir B. Brodie (case 50), and Mr. Mayo (Med.-Chir. Trs. vol. xix, pp. 63-4,) each adduces a case in which they affirm that cartilage of incrustation was distinctly seen to be sensibly vascular, and when Mr. Liston, (Med.-Chirurg. Trs. vol. xxiii, p. 92 et seq.) professes to have determined the point in the affirmative by microscopical examination of the injected vessels, it is difficult to deny the fact, either on theoretical grounds, or on the strength of negative researches, however numerous. In the present state of our knowledge, the weight of evidence goes to establish that ulceration of cartilage by the action of its own vessels, is, at the least, an exceedingly rare occurrence; and the direct proof of its becoming vascular in disease of the joints, has been obtained by but a few observers, and in a few cases only; while it is at the same time indisputable that cartilage is frequently removed by the action of the adjacent tissues, without itself having any active participation in the process. We shall hereafter have occasion to remark on some of M. Richet's views respecting the pathology of the bone.

*Experiments on the Joints.* M. Bonnet adds, as an appendix to the chapter on pathological anatomy, an account of the effects produced "*by the forcible injection of liquid into the joints,*" (p. 50,) effects which, though entirely artificial, greatly elucidate, he thinks, important points connected with the symptoms and treatment of diseases of the joints. We scarcely think those experiments so fruitful in these respects as their author does, but they are certainly sufficiently interesting and important to require concise but accurate notice.

In injecting the joints, M. Bonnet, in the case of the hip, the knee, and the shoulder, lessened the weight of the limb by amputating it a little below the joint, and threw in, through a perforation in one bone (which was fixed), an injection which became solid on cooling, and he thus obtained an exact cast of the cavity of the articulation at its maximum distension, or in the event of the capsule being ruptured, could trace the exact course of the effused fluid.

The general effects of forcibly injecting a joint, are, 1st, To make the bones assume a determinate position, always the same in the same joint whatever was its position previously. 2dly. To enlarge the cavity of the joint to its maximum capacity. 3dly. To separate the bones, and always interpose a layer of the injection between the entire extent of their articular surfaces, contrary to the opinion of Sabatier, Boyer, Larrey, &c. That liquid in a joint, say the hip, must simply distend the capsule, and accumulate round the bones without being interposed between them. 4thly. To render the joints motionless till a portion of the fluid escapes. 5thly. When the fluid is pushed too forcibly, to rupture the capsule of the joint where it is thinnest and least supported.

The cause of the motion communicated to, and of the position assumed by, the bones of a joint when it is injected, is the interposition of the injected liquid between the articular surfaces and the unequal resistance of the ligaments; for the interposed fluid tends to separate the bones, and if they are kept at a given degree of approximation at one point, and free to separate at others, the point at which further separation is prevented is a pivot on which they move. The anatomical character of a joint, therefore, determines the direction of its motion when it is injected. In the orbicular joints, if the capsule is denser and less distensible at any one part than elsewhere, that part is the centre of motion. In the ginglymoid joints the two lateral ligaments simultaneously resist the separation of the bones, and therefore if the extent of the articulating surfaces is the same before and behind the lateral ligaments, the bones will assume the straight position, but if there is a greater extent of the articulating surfaces, either before or behind the lateral ligaments, the pressure of the liquid will be greater on the more extensive surface, and consequently the joint will be flexed in the direction opposite to that surface. (vol. i, pp. 50-64.) Thus the *knee*, when injected, assumes the semiflexed position at an angle with the femur somewhat greater than a right one; the lateral ligaments being situated nearer the back than the front of the joint. (vol. ii, p. 152.) In the case of the *hip*, the femur is flexed so as to make an angle of about sixty degrees with the abdomen, and is at the same time abducted and rotated outwards, because the capsule, being scarcely extensible anteriorly, and externally, and considerably so inferiorly and internally, the head of the femur can be separated much further from the acetabulum in the latter situation; but the motion in which the inner and lower part of the head of the femur is separated more than its outer and anterior part from the acetabulum, is just that during which the shaft of the bone is carried forwards and outward, or, in other words, is flexed and abducted. The rotation of the femur outwards arises from the direction of the fibres of the capsular ligament, being oblique from above downwards, and from without inwards, and when the capsule is distended, they tend to become straight, and in so doing exert a traction on the bone which rotates it outwards. Moreover, the shape of the articulating surface of the femur is such, that it cannot be flexed and abducted without a tendency to rotation outward. (vol. ii, p. 266.) In the *ankle,* the foot is *very slightly* extended on the leg. In the *shoulder* the humerus is abducted at an angle of about thirty-five degrees, with the side of the thorax, and carried forward in an arc of a circle of about fifteen degrees, because the capsule is least distensible, and shorter at its outer and upper part. In the *elbow* the fore-arm is flexed to nearly a right angle with the humerus, in a position intermediate between pronation and supination. In the *wrist* the hand is always brought straight with the fore-arm.

As forced injection brings a joint into a determinate position, we might expect that those experiments would explain the position assumed by joints in diseases accompanied with effusion into their cavities; but it is only when the effusion is very rapid, as well as abundant, that it can produce this physical effect of forced injection; and even then the effect would not be entirely physical, for in the position in question the capacity of the joint is at its maximum, and consequently the tension of the fluid on the capsule the least possible, but this is precisely the posture the patient would instinctively select to diminish pain. Whenever effusion into a joint occurs somewhat slowly, the capsule yields to the progressive distension, and the liquid not being interposed between, and therefore not tending to separate the bones, they are not forced into the position which leaves the greatest space between them. M. Bonnet somewhat superfluously applies those experiments as grounds for insisting on the necessity of perfect rest in penetrating wounds of joints. As the capacity of a joint varies with its position, it must be alternately increased and diminished during motion; and when increased, as part of its parietes are rigid, there is a tendency to a vacuum; and, when the joint is open, air passes in, (the prejudicial influence attributed to which by M. Bonnet, we shall hereafter consider;) when a joint, therefore, is wounded, we should keep it perfectly motionless in that position in which its capacity is least; and, by a fortunate coincidence, that position is the one in which an anchylosed limb is the most useful, being extension in the hip, knee, and wrist, and flexion in the ankle and elbow. (vol. i, pp. 262-8.)

*Position of the limbs in diseased joints.* M. Bonnet discusses the causes and effects of a faulty position of the limbs in diseases of the joints in the chapter on "*Etiology,*" but this is a more convenient and seems also a more suitable place to consider this subject, to which

M. Bonnet attaches the greatest importance. He considers that a faulty position of the limb exerts the most powerful influence on the progress of every disease of a joint, and that the re-establishment of a correct position is one of the [often the most] powerful remedial agents at our command. In a few exceptional cases, a bad position of the limb may not aggravate the evil, but, as a general rule, it augments inflammation and pain, by exerting a violent and continued tension on the synovial membrane, the ligaments, and the soft parts generally, and leads to spontaneous luxations, which are simply the result of a vicious posture of a joint whose ligaments are weakened or destroyed. (vol. i, p. 89.)

The causes of the position assumed by a limb in disease of a joint, are, 1st. The physical effects of an accumulation of liquid in the synovial cavity. 2dly. The weight of the limb, and the pressure of external objects, as the bed-clothes, &c. on it. 3dly. The necessity experienced by the patient of keeping the affected joint as motionless as possible. 4thly. The active or passive contraction of the muscles. The first of these causes has been already alluded to, the others shall be presently noticed when illustrating the mechanism of the deformity in some of the individual joints.

Any position of a joint is injurious,—1st, which causes continued distension of the soft parts on one side of the joint,—for the synovial membrane, ligaments, &c. are uniformly found most diseased on the side so distended; 2dly, which determines considerable and continued pressure on any part of the bones of the joint,—for the bones and cartilages are more extensively absorbed and ulcerated where such pressure exists; and, 3dly, which tends to produce spontaneous luxations, (vol. i, pp. 78-85.) The influence of such positions will be best estimated by referring to some particular joint.

In disease of the *knee* the patient may lie on his back with the limb extended and lying on its posterior surface, but usually it is rotated outwards by its own weight, a rotation increased by the weight of the bed-clothes when once commenced. But this posture is unsteady, and is therefore seldom adopted; the patient seeks a more fixed position, and for this purpose turns more or less on either side, and at the same time flexes the leg on the thigh, and the thigh on the pelvis, in order to give the limb an extended base of support. The same thing indeed is commonly done in health when lying on the side, with the lower extremities extended; it is difficult to keep the body motionless without an effort, which ceases to be necessary when the limbs are flexed. If the patient lie on the diseased side, the knee is everted; if he lies on the sound side, it is inverted. When the flexion is once thus established, it tends to cause displacement in the following manner:—The leg, when extended on the thigh, enjoys neither lateral nor rotatory motion; but if it is flexed on the thigh at any angle of from forty to one hundred degrees, it is easily rotated both outwards and inwards; because in the extended position the whole of the articulating surface of the tibia is in contact with that of the femur, and the tibia cannot rotate on its axis without one of its condyles being carried backwards against the posterior ligament, which resists the motion; but in the semi-flexed posture, the centre only of the tibia is in contact with the femur, and the posterior ligament being relaxed, offers no resistance to rotation; spontaneous luxation of the tibia, therefore, especially that accompanied with rotation, is favoured by the semi-flexed posture. But greatly the more serious mischief results from the pressure generally exerted on the joint when in this position. If, indeed, the limb lies on its *entire* outer surface, there is neither distension of the ligaments, &c. nor tendency to spontaneous luxation. But lateral decubitus is seldom complete; and, when the limb is everted, it usually bears chiefly on the outer edge of the tarsus, and of the lower part of the leg, while the knee is at the same time imperfectly supported. The tibia consequently tends to make with the femur an angle salient externally; and, therefore, the external lateral ligament, and the soft parts generally of the outside of the joint, are necessarily distended. Furthermore, the lower part of the leg is carried by its own pressure on the bed forward and inwards, whence its upper portion is impelled in the reverse direction, or backwards and outwards. All those causes, therefore, concur to produce a partial luxation backwards, outwards, and with rotation outwards. When the semi-flexed limb is *inverted,* the effects of the position are different. The limb is then supported on the entire inner edge of the foot, and there is then no tendency to luxation with rotation, or to luxation backwards. The lower part of the leg is carried directly outward, whence the tibia tends to make with the femur an angle salient internally, and re-entrant externally, and the internal lateral ligament, &c. being thus put on the stretch, it might be expected that the tibia would tend to become luxated directly inward. But M. Bonnet has not observed this in three cases, which he has seen since his attention was particularly directed to the relation between spontaneous luxations and the position of the limb, in which the leg rested on the internal border of the foot, there was no luxation, but the tibia and the femur were absorbed externally where they forcibly pressed against each other, and a considerable hollow existed in this situation when the limb was straightened. (vol. ii, pp. 156-62.)

In diseases of the *hip,* the thigh is never extended on the pelvis, as it is when we stand in a perfectly vertical posture. The femur is always flexed on the pelvis, usually at an angle of about 150 degrees, and may or may not also deviate to either side. Hence the thigh may assume three positions in relation to the pelvis. 1st, Flexion without lateral deviation, which is extremely rare; 2dly, Flexion with abduction, which is always coupled with rotation outwards, and occurs frequently; 3dly, Flexion, with abduction, which is the most frequent, and always coincides with rotation inwards. These are the only positions that occur in diseases of the hip, but the flexion of the thigh may sometimes be easily overlooked; thus, when the patient lies on the diseased side, the inferior extremities may be extended on the bed, but the pelvis is then flexed on the femur; in other words, the axis of the pelvis, and the axis of the femur, form an angle open anteriorly, just as when the pelvis is fixed, and the thigh flexed on it.

As to the cause of the flexion of the femur, it is sometimes at least partly referrible to the cavity of the joint being most capacious in this position, whence the diminution of pain consequent on the relaxation of the capsule of the joint occasionally causes it to be selected; but M. Bonnet thinks this position is assumed chiefly because it is always the natural position of the thigh when at rest in the recumbent position. When lying on the back, the trunk is raised by pillows, and therefore flexed on the thigh, and in decubitus on the side, the thigh is flexed to give a sufficient base of support to the body. But why is the thigh, when flexed, sometimes everted, sometimes inverted, and sometimes neither? M. Bonnet attributes these differences chiefly to the position in which the patient habitually lies. During dorsal decubitus, if the ham is supported by a pillow, the thigh may be simply flexed directly forwards; but if the limb is not supported behind, or if the patient lies on the affected side, it is everted; on the contrary, it is inverted by its own weight when the patient lies on the sound side. But if the mode in which the patient lies alone determined the inclination of the thigh outwards or inwards, adduction should only occur when the patient lay habitually on the sound side, but it occurs also when the patient lies on the diseased side; this is sometimes caused by the relation of the femur to the pelvis having been previously established while the patient lay in another position; but sometimes it arises from this:—as has been already stated, rapid and copious effusion into the hip-joint abducts and rotates the thigh outwards; but when the capsule becomes perforated, the disease has often made much progress at the inner part of the joint and among the soft parts external to it, and the patient inverts the thigh to relax the soft parts in that situation. This explanation, M. Bonnet says, tallies perfectly with facts. Usually at the commencement of disease of the hip joint, the femur is flexed, abducted and rotated outwards, and at a more advanced stage is flexed, adducted, and rotated inwards.

As to the *effects* of those positions, *slight* direct flexion of the femur is the most favorable posture, for then the capsule is nowhere distended, nor does the head of the femur press unequally on the cotyloid cavity; neither is there any tendency to luxation; and anchylosis, if it occurs, does so in the most favorable position. Considerable flexion is, however, injurious, as the head of the femur then distends the capsule posteriorly; and in the event of anchylosis, when the patient attempts to walk, the hips are projected back, and the trunk arched forward, as during progression the thigh must be nearly vertical, and the trunk must assume a position corresponding to the deformity of the limb. In flexion, with abduction, and rotation outwards of the femur, the evils just mentioned are aggravated, the capsule is distended internally and anteriorly, whence there is a tendency to dislocation into the foramen ovale, if the flexion approaches a right angle, or on the pelvis if the flexion is less, events, however, which very rarely occur. In flexion of the thigh, with adduction and rotation inwards, the head of the femur bears against the posterior and upper part of the capsule of the joint, and of the cotyloid cavity, and spontaneous luxation is greatly facilitated by the deficiency of the brim of that cavity in that situation. (vol. ii, pp. 267-76.)

It is unnecessary to go through M. Bonnet's account of the mechanism and effects of position in the other joints, as we shall hereafter state his practical deductions from his views with respect to each joint. We shall for the present only add that M. Bonnet lays down, as an invariable rule of practice, that "whatever is the disease of a joint which we have to treat, if the diseased limb is in a vicious posture, it should be reduced to a favorable one." (vol. i, p. 119.) This rule is not confined to chronic diseases, but is applied to every disease and every stage of every disease. Thus, in an acute inflammation of a joint (say the knee), he objects to the practice usually followed of leaving the limb in the position selected by the patient, and not attempting to straighten it till the inflammation has subsided, and on the contrary, inculcates that the limb should be at once, 1st, brought into a favorable position, and 2dly, then kept immoveable in that position by means of a suitable apparatus. These are the two great indications, according to M. Bonnet, to be fulfilled in the surgical treatment of diseases of the joints. In acute inflammation, a vicious position of the limb keeps the soft parts of the joint on the stretch; by straightening the limb, this painful distension is removed, and the inflammation and pain are allayed. The cases he gives, he says,

"Will demonstrate better than any reasoning the justness of his principles. They will leave no doubt of this truth, that when the most energetic antiphlogistics have completely failed in an inflammation of the knee, and the malady is becoming aggravated, straightening the limb and then keeping it immoveable by means of a suitable apparatus, is sufficient to arrest the progress of the disease, to cause cessation of the pain, and progressive amelioration." (vol. ii, p. 203.)

M. Bonnet acknowledges that this method occasions severe pain during the entire time necessary for straightening the limb; a period which is but short if the disease has not lasted for more than a month, but extends to two or three days if the malady has existed for two or three months. But once the limb is straightened and rendered motionless, the amelioration is immediately experienced. In upwards of twenty cases, he met two only which were not amended; those were cases of inflammation of the wrist, which he kept pronated, and he has since ascertained that the failure arose from having adopted this position, as the wrist should be kept in a position intermediate between pronation and supination.

*The effects of protracted immobility of joints* will be here properly noticed, both because of the pathological changes induced thereby, and because M. Bonnet founds thereon a rule of practice which he considers of very great value and importance.

It has long been admitted that protracted rest is capable of producing stiffness and some other alterations of the joints; but though the subject has been alluded to by J. L. Petit, Hunter, Boyer, Cruveilhier, Velpeau, Kunholtz, Malgaigne, and Vidal de Cassis, it has been more especially investigated by M. Teissier, whose researches go to establish that protracted immobility may cause—1st, effusion of blood and of serum into the cavity of a joint; 2d, vascularity and formation of false membranes on the synovial membrane; 3d, alterations of the cartilages; 4th, anchylosis.

Cloquet and Sanson both mention 'a local scurvy,' produced by long-continued rest during the treatment of fracture, especially the violet spots that not unfrequently appear on the skin. M. Teissier was the first to notice sero-sanguineous effusion into the joints under the same circumstances. Having for a considerable period taken every opportunity of examining all the joints of limbs kept long motionless, whether during the treatment of fracture or by paralysis, he almost constantly found all the joints, even those most remote from the injury, containing bloody serum, or even blood almost unmixed, and in one instance clotted. Similar effusion also often existed in all the soft parts external to the joint. This effusion always coexisted with vascularity, especially of the fringed folds of the synovial membrane, which were swollen and red. False membranes were occasionally, but not often, found along with the foregoing alterations, and in every instance in which M. Teissier did find them they were already penetrated by vessels, and adhered to the cartilages. The cartilages sometimes presented an uniform or a punctated redness. When the cartilages were not eroded, the redness occurred as ecchymosed maculæ; when, on the contrary, they were eroded, it was punctated, and in one instance resembled arborescent vascularity, but this, M. Bonnet says, is the result merely of the cartilage imbibing the blood; for though redness by imbibition is uniform when the cartilage retains its natural structure, its surface becomes villous when the imbibition is punctated. Anchylosis is of course a remote result of perfect rest, and, so far as is known, is established by the false membranes adhering, and becoming cellular or fibrous. M. Bonnet gives several cases in which, after fracture of the shaft or of the neck of the femur, the foregoing alterations (excepting anchylosis) were found on dissection, not only in the knee but in the ankle. And M. Velpeau has published two cases which prove the possibility of anchylosis by cellular or fibrous union being caused by long-continued rest. The circumstances that favour the occurrence of those changes in joints when kept motionless are: 1st, the duration of immobility; 2d, old age; 3d, the whole body being kept at rest; 4th, a feeble or cachectic constitution. Whence they are less likely to occur in young, healthy, well-fed persons, and in those fractures, as of the upper extremities, which do not require confinement to bed, or do not take long to consolidate. M. Bonnet thinks it impossible, in the present state of our knowledge, to explain the occurrence of these alterations. From their nature they might be considered inflammatory, and supposed to depend on extension of inflammatory action from a fracture, or on some injury received at the time of the accident; but they occur in the joints most remote from the fracture, and also have been observed where the limb was motionless in consequence of paralysis. Moreover, in cases where the neck of the femur was fractured, the knee and ankle were considerably affected, while the hip was very little so; a result explained by the difficulty of maintaining the hip motionless, while the knee and the ankle are easily kept so. Finally, the alterations arising from immobility are not purely inflammatory; the characters of the sanguineous effusion are analogous to those of scurvy. M. J. Guerin attempts to account for these alterations thus: we have seen that the capacity of a joint varies during motion; in certain motions, therefore, there is a tendency to a vacuum produced in the joint, whence a suction is exerted on its parietes, which excites the exhalation of fluid; and thus he attributes to atmospheric pressure an important influence on the mechanism of serous exhalation. But when a joint is at rest, there is an equilibrium between the external and internal pressure, and therefore no suction and consequent suppression of the synovia; and M. Guerin thinks that the exhalation of the synovia being arrested, the fluids stagnate in the vessels, which leads to congestion and other morbid changes. This explanation, M. Bonnet objects, supposes that the synovia is diminished in quantity; but the very contrary is the fact. (vol. i, pp. 67-78, and vol. ii, pp. 133-6.) We may here anticipate so far as to say that M. Bonnet deduces from the researches of M. Teissier, from the experiments of M. Guerin on the influence of atmospheric pressure on the exhalation of synovia, and from his own clinical experience, that motion exerts a most powerful influence in modifying the function and morbid condition of a joint; and that a joint should not be kept perfectly motionless for a considerable period in any disease, except in acute inflammations attended with considerable pain, in wounds of joints, and in those chronic cases in which a cure can only be expected from the establishment of anchylosis. (vol. i, p. 132.) But how are we to determine (suppose in a case of acute inflammation becoming chronic,) when immobility is to be replaced by motion? M. Bonnet gives no rule on this point beyond expressing an opinion that, unless some contra-indication exists, rest should not be prolonged beyond six weeks; but he quotes, without expressing any opinion as to its value, the following method for obviating this difficulty, proposed by M. Malgaigne in the Journal de Chirurg. (Oct. 1844):

"A certain method of diagnosis is pressure on the diseased joint, but not on every part of it indifferently, for experience shows that the pain has a place of election in each joint. Thus, in the shoulder, pressure posteriorly sometimes, but rarely, occasions pain; the place of election is anteriorly; elsewhere pressure may be made with impunity. In the elbow the place of election is over the head of the radius; in the hip, at the posterior part of the head of the femur, behind the great trochanter. If the pressure on the head of the humerus, anteriorly and posteriorly, on the anterior and posterior part of the head of the femur, and on the head of the radius, causes no pain, we may pronounce that arthalgia of the shoulder, of the hip, or of the elbow, has terminated, that the period for perfect rest has terminated, and that danger from motion has ceased; and then we should fearlessly encourage the patient to bear with fortitude the pain which he must endure on the very threshold of the second period of the treatment." (Vol. i, pp. 422-3.)

*Experiments on mechanical lesions of the joints.* Some account of M. Bonnet's experiments on the injuries resulting from forced motions of the limbs may naturally find a place here. It would, however, be superfluous to give a summary of all his experiments,—which, by the way, are detailed at most wearisome length,—both because many of the results are unimportant, and also because they chiefly bear on the history of fracture and dislocations, subjects, except so far as these experiments go, excluded from M. Bonnet's work. We shall, however, in order to give a general idea of M. Bonnet's researches, refer to a few points which may be interesting, either because of their novelty, or their possible practical application.

Fractures of the malleoli of the ankle, and of the inferior extremity of the radius, from violent motion impressed on the foot, or on the hand, are perfectly well known, but it is not so generally understood that analogous motions of the knee, the elbow, the shoulder, and the hip, may also cause fractures of the shafts, or of the articular extremities of the bones.

In the adult, when the leg is rotated violently on the thigh, as a general rule the knee remains intact, and the tibia is fractured about its centre, and the fibula close to its superior articulation; those fractures are always very oblique, but no relation could be traced between the direction of the obliquity and of the rotation. The tibia is first fractured, and the fibula occasionally escapes, from the motion allowed of in its superior articulation, and from its elasticity permitting of a certain extent of torsion. The fractures of the fibula also may readily escape detection, the fragments being held together by the periosteum, and by the fibrous and aponeurotic expansions which cover the bone at the situation of the fracture. In children the results are usually the same, but in two out of eight experiments, the femur, and not the tibia, was fractured. In one case the femur was twisted just above the condyles, so that the external surface of the shaft corresponded to the anterior surface of the condyles. In the second case there was a very oblique fracture of the lower third of the thigh; the periosteum was only partially torn, and intervened between the fragments when an attempt was made to cross them. We may here mention that he scarcely ever, in his experiments on young subjects, detached the epiphysis; the bone was almost constantly broken immediately above the epiphysal cartilages; the deformity was very slight, and the fragments were not completely separated, being in a manner locked by numerous asperities, and always retained by some untorn portion of periosteum; sometimes indeed the periosteum was intact, and had to be divided to expose the fracture.

Generally, as already said, the knee is uninjured when the leg is forcibly rotated on the thigh, but in one experiment the tibia was partially luxated on the femur, and the appearances in this case explain, M. Bonnet thinks, the accident described by Sir A. Cooper and Mr. Key as dislocation of the semilunar cartilage of the knee. In this experiment the foot was suddenly rotated outwards while the leg was bent to a right angle with the femur, and immediately a peculiar sensation of something having slipped (*d'un soubresaut particulier*) was felt. The foot remained everted and the leg flexed at an angle of about 45 degrees with the thigh; the internal condyle of the tibia projected a little inward and forward beyond the internal condyle of the femur, and the head of the fibula was carried backwards and inwards. On extending the leg gently on the thigh, another slight slip was felt and the bones resumed their natural position; dissection showed that none of the ligaments or of the muscles were torn. M. Bonnet raising the patella to inspect the cavity of the joint again rotated the leg outwards, and saw that the slip or jolt was occasioned by the internal condyle of the femur passing behind the semi-lunar cartilage which was thus thrust forward on the internal articulating surface of the tibia, the capsule of the joint remaining unruptured; the external condyle of the femur was carried very slightly forward but remained in contact with the external semi-lunar cartilage. The joints of the subject experimented on, were, M. Bonnet says, very lax. The appearances in this experiment agree very well with the account of the cases termed dislocations of the semilunar cartilage, as regards the way in which the displacement was occasioned and the mode of remedying it, but differ in the very obvious deformity observed in M. Bonnet's experiment; still we think the experiment throws considerable light on the affection described by Key and Cooper, which, many surgeons suggest, were not examples of displacement of the semi-lunar cartilage but probably cases of foreign bodies in the knee. M. Bonnet, we have seen, considers that the affection in question, is a partial dislocation of the condyle of the femur, permitted by relaxation of the ligaments of the joint—a view already taken of the affection by Malgaigne; but if the attachments of the semilunar cartilages, both to the tibia and to the capsule of the joint, are very lax, so as to allow them to slide on the tibia, (which is proved by dissection to occur,) then the cartilage may be pushed forwards by the condyle of the femur, instead of the condyle of the femur slipping behind it, as occurred in M. Bonnet's experiment.

To enter into a further account of M. Bonnet's experiments on mechanical lesions of the joints, would lead us too far from the proper subjects treated of in his work; but other occasions will occur on which we can notice them, so far as is necessary, in connexion with fractures and dislocations, to which, as has been already mentioned, they principally refer; and as it would be impossible to examine those parts of M. Bonnet's work which relate to the etiology, symptoms, and treatment of diseases of the joints within the limits now at our command, we shall conclude for the present, and resume the subject in our next Number.

